# Case Report: Acute Eosinophilic Myocarditis With a Low-Flow Heart Failure With Preserved Ejection Fraction Phenotype

**DOI:** 10.3389/fcvm.2021.678973

**Published:** 2021-06-23

**Authors:** Hiroto Aota, Hiroyuki Yamamoto, Jun Isogai, Kyoko Imanaka-Yoshida, Michiaki Hiroe, Takahiro Tanaka

**Affiliations:** ^1^Department of Cardiology, Cardiovascular Center, Showa General Hospital, Tokyo, Japan; ^2^Department of Cardiovascular Medicine, Narita-Tomisato Tokushukai Hospital, Chiba, Japan; ^3^Department of Radiology, Asahi General Hospital, Asahi, Japan; ^4^Department of Pathology and Matrix Biology, Mie University Graduate School of Medicine, Tsu, Japan; ^5^Department of Cardiology, National Center for Global and Medicine, Tokyo, Japan

**Keywords:** heart failure, HFpEF phenotype, CT-guided EMB, corticosteorids, AEM

## Abstract

Eosinophilic myocarditis is a rare subtype of myocarditis characterized by myocardial eosinophilic infiltration, and it is potentially fatal if left untreated. Although endomyocardial biopsy (EMB) is a cornerstone for the histological diagnosis of acute eosinophilic myocarditis (AEM), as it is an invasive procedure and has a low diagnostic accuracy, the diagnosis of AEM with hemodynamic instability remains challenging. We describe a case of AEM presenting as low-flow heart failure with preserved ejection fraction (HFpEF), with rapid progression to cardiogenic shock. The constellation of peripheral eosinophilia, increased left ventricular wall thickness, and HFpEF raised the suspicion of AEM. Contrast-enhanced computed tomography (CT) scan revealed heterogeneous hypoenhancement localized in the basal-to-mid septal and mid anterolateral walls of the left ventricle, strongly suggestive of acute inflammation. Based upon these findings, we performed CT-guided EMB, which lead to a definitive diagnosis. Subsequent high-dose corticosteroids allowed a rapid and dramatic recovery and normalization of cardiac structure and function. This case highlights the clinical importance of assessing AEM as a rare cause of HFpEF and the usefulness of CT-guided EMB in patients with hemodynamic instability.

## Introduction

Eosinophilic myocarditis (EM) is a rare inflammatory disorder characterized by eosinophilic infiltration of the myocardium, frequently accompanied by peripheral eosinophilia (1). EM, which occurs in ~0.5% of the autopsy cases, is most frequently associated with heart transplantation, identified in about 3% of explanted heart specimens (2). However, its exact incidence remains unknown. The diagnosis of EM is equally frequent among both sexes, especially in those aged around 40 years (3). EM advances through three chronological processes: acute necrosis, thrombosis, and fibrosis, which may overlap. EM presents with various clinical manifestations depending on the degree of eosinophilic infiltration of the myocardium, which ranges from asymptomatic to heart failure, acute fulminant myocarditis, malignant arrhythmias, intracardiac embolism, and restrictive cardiomyopathy (4). Among them, acute eosinophilic myocarditis (AEM) progresses rapidly, resulting in fatal outcomes if left untreated. However, AEM is extremely difficult to diagnose owing to its nonspecific signs and symptoms.

## Case Description

**Figure 1:**
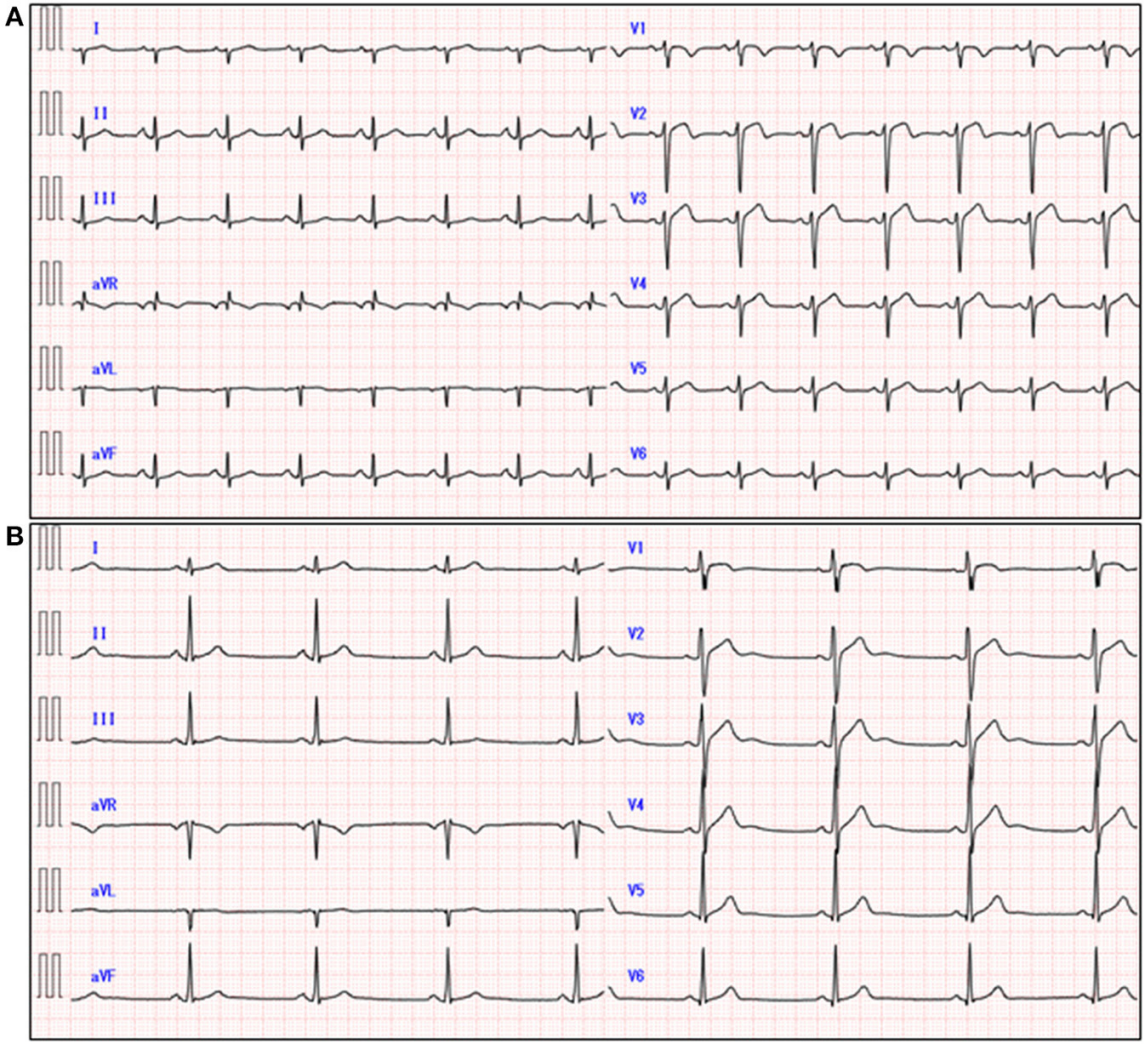
Electrocardiogram at admission **(A)** and on day 11 **(B)**.

**Figure 2:**
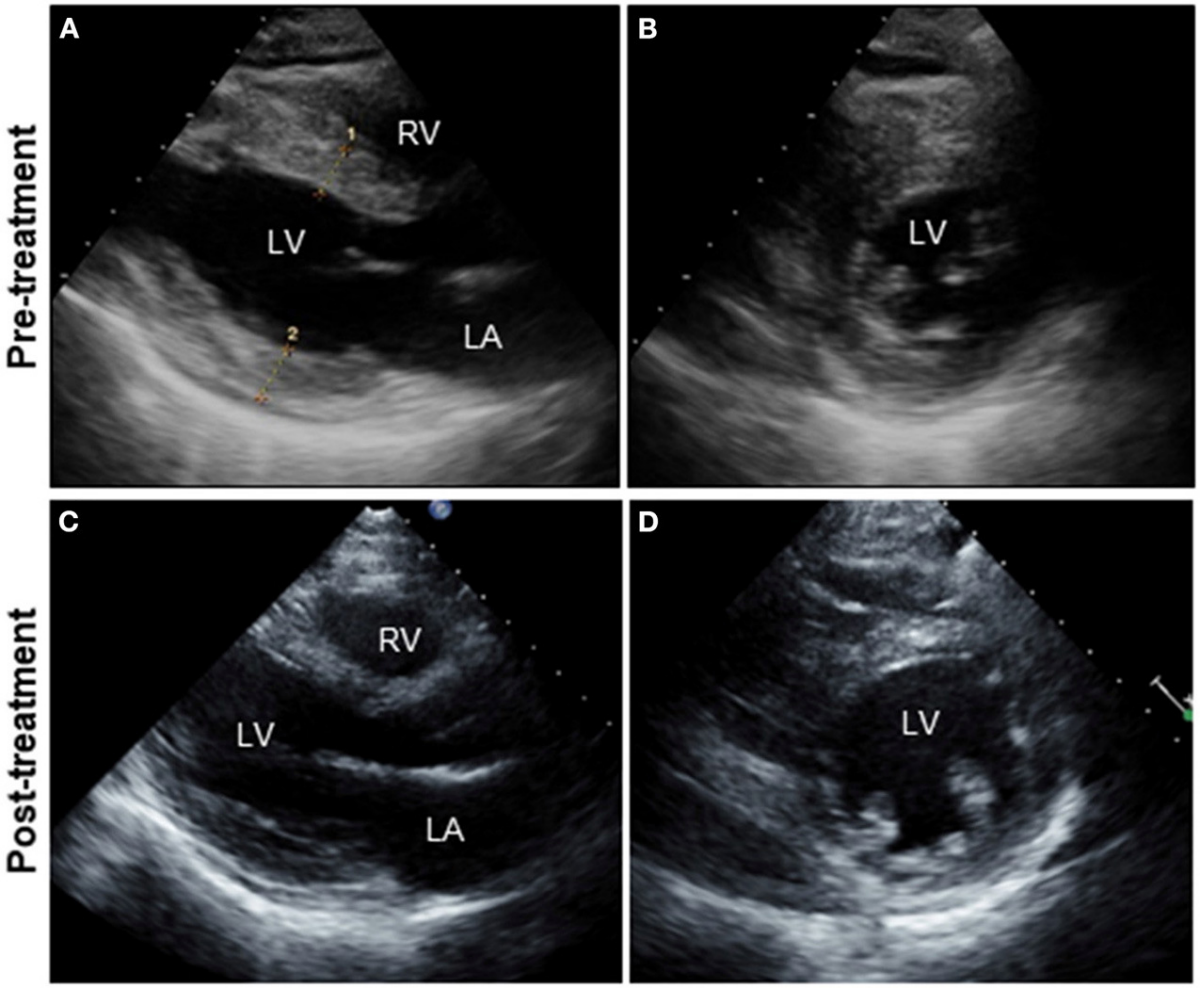
Serial TTE. Parasternal long-axis **(A,C)** and short-axis views **(B,D)**. TTE on admission showing diffuse and symmetrical LV wall thickening (13mm), decreased cavity size (LVDd: 39 mm) with preserved LVEF (52%), and pericardial effusion **(A,B)**. Follow-up echocardiography on day 4 after intravenous pulse steroid therapy reveals a rapid and dramatic decrease in LV wall thickness (7 mm) and concomitant increases in LVDd (45 mm) and LVEF (72%) **(C,D)**. The improvement in pericardial effusion can be noted. TTE, transthoracic echocardiography; LA, left atrium; LV, left ventricle; RV, right ventricle; LVDd, left ventricular end-diastolic diameter; LVEF, left ventricular ejection fraction.

**Figure 3:**
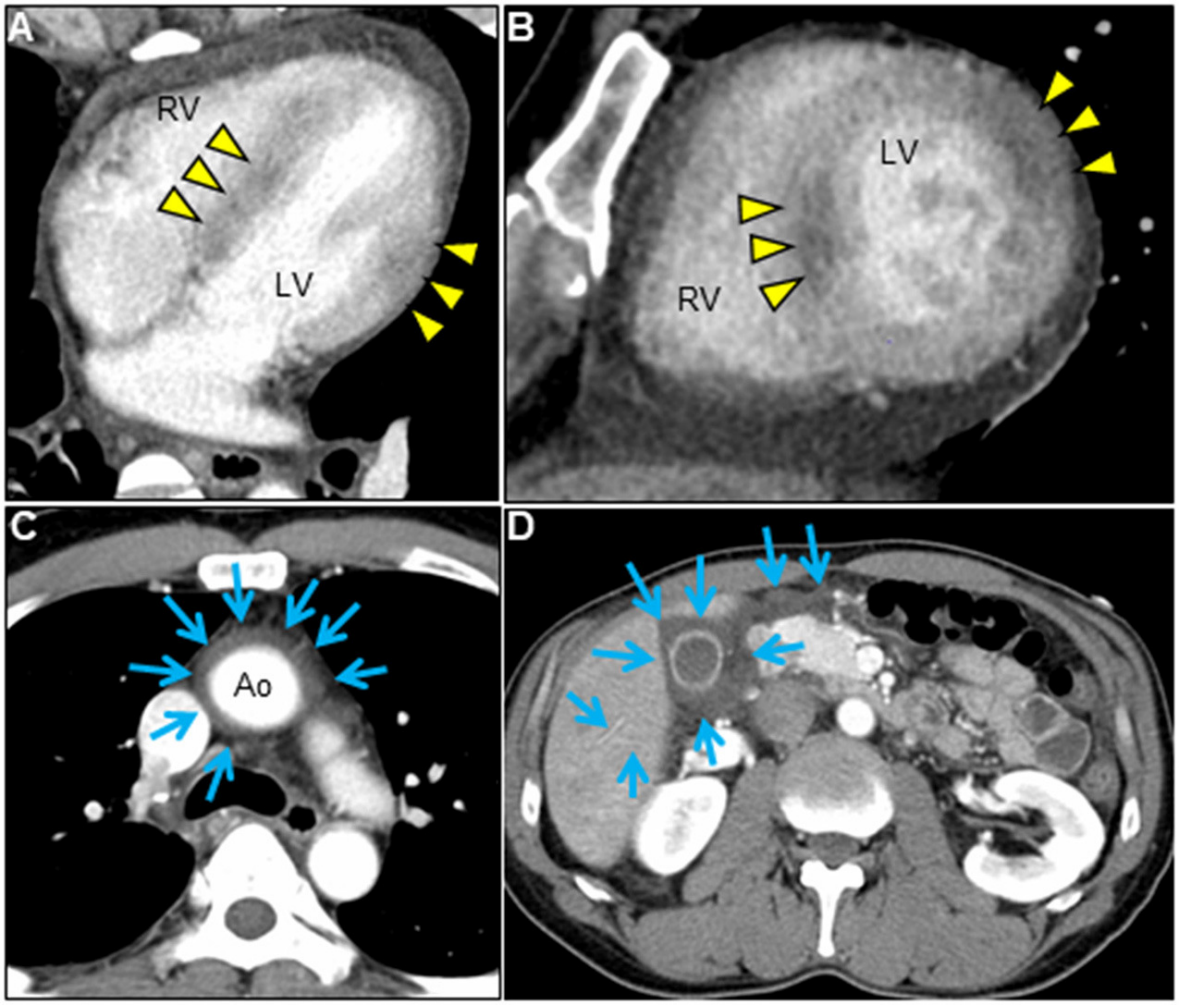
Chest/abdominal contrast-enhanced CT images. Axial **(A,C,D)** and sagittal **(B)** views. Contrast-enhanced CT reveals LV wall thickening with pericardial effusion, decreased myocardial enhancement of both mid-layer in the basal-to-mid septal wall and transmural myocardium in the mid anterolateral wall (**A,B**, arrowheads), and fluid accumulation in pericardial sinuses (**C**, arrows). Airless low-attenuating areas in the region of gallbladder wall thickening and around the hepatic round ligament and periportal space, indicating marked systemic edema (**D**, arrows). CT, computed tomography; Ao, aorta; LV, left ventricle; RV, right ventricle.

**Figure 4:**
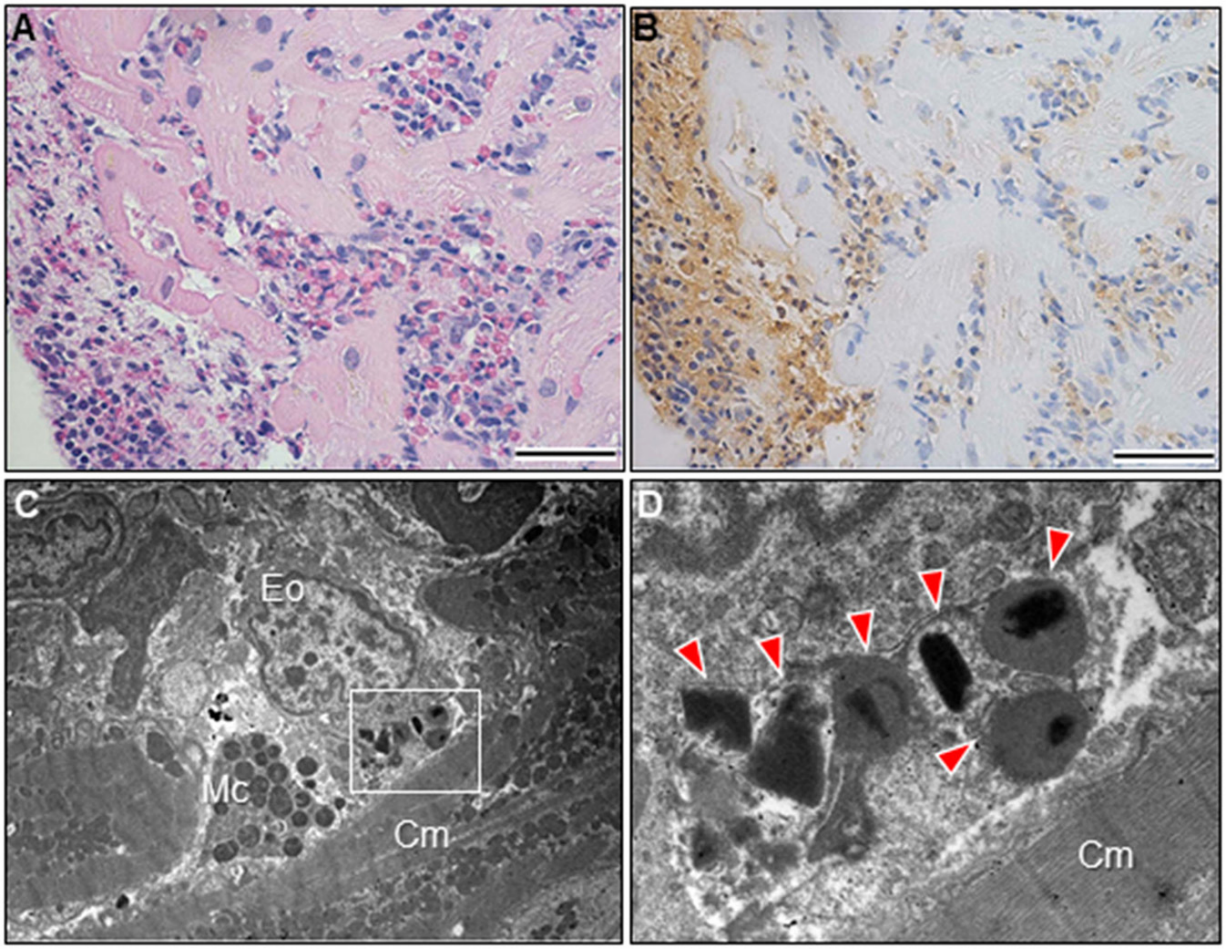
Endomyocardial biopsy findings. Photomicrograph with hematoxylin and eosin stain **(A)**. The prominent eosinophilic infiltrate with degranulated eosinophils and surrounding edema and the layers of thickening endocardium can be noted. Photomicrograph with immunostaining against the eosinophilic cationic protein **(B)**. Bars: 50 μm. Transmission electron microscopy shows myocardial interstitial infiltration by degranulated eosinophils and mast cells in close proximity to cardiomyocytes **(C)**. A high magnification view of the region outlined by the box in Figure 4C **(D)**. Eosinophilic granules can be noted (arrowheads). Cm, cardiomyocyte; Eo, eosinophil; Mc, mast cell.

A 51-year-old previously healthy man, with no significant past medical history except for hay fever, was referred to our hospital for evaluation of chest discomfort and recurrent presyncope. He has begun to feel the above symptoms the day before admission. On admission, the patient was afebrile with a pale complexion. His vital signs were as follows: blood pressure 86/60 mmHg, heart rate 89 beats/min, and respiratory rate 18 breaths/min. Mild bilateral leg edema was observed. The electrocardiogram (ECG) revealed sinus rhythm, low voltage limb leads, inverted T-waves in V1, 2, and poor R-wave progression (Figure 1A). Chest radiography revealed mild congestion without any signs of cardiomegaly. Laboratory testing revealed a white blood cell count of 6,800/μL with mild eosinophilia (absolute eosinophilic count: 884/μL; 13% in differential), mildly impaired hepatic function tests, elevated levels of C-reactive protein (0.99 mg/dL, reference: <0.5 mg/dL), cardiac troponin T (0.620 ng/mL, reference: <0.014 ng/mL), and brain natriuretic peptide (183 pg/mL, reference: <18.4 pg/mL). Echocardiography demonstrated symmetrically increased left ventricular wall thickness (ILVWT) of 13 mm with small left ventricular (LV) cavity size, preserved systolic function with an ejection fraction of 52%, and pericardial effusion (Figures 2A,B and Supplementary Videos 1, 2). Doppler profile assessment measured a low LV outflow tract velocity time integral (13.8 cm, reference: >15 cm), suggesting a low stroke volume. Thus, a tentative diagnosis of acute heart failure with preserved ejection fraction (HFpEF) of wet-cold phenotype was made. The constellation of peripheral eosinophilia, ILVWT, and HFpEF inclined us toward strongly suspecting AEM. A subsequent contrast-enhanced chest/abdominal computed tomography (CT) scan showed diffuse LV wall thickening with heterogeneous non-ischemic hypoenhancement, suggesting acute inflammatory edema (Figures 3A–C). It showed characteristic hypoenhanced areas in the basal-to-mid septal and mid anterolateral walls of the LV, along with revealing fluid accumulation in both the pericardial sinuses and pericardium. Moreover, we observed gallbladder wall edema and fluid accumulation around the hepatic round ligament and periportal space, suggestive of rapid systemic congestion caused by heart failure (Figure 3D). Given the fact that these abnormal findings were extensively observed simultaneously, it was highly likely that the condition had progressed rapidly. The patient's condition then rapidly progressed to cardiogenic shock, requiring vasopressor and inotropic support with noradrenaline (0.05 mcg/kg/min) and dobutamine (2 mcg/kg/min) infusions. Coronary angiography was unremarkable, and an intra-aortic balloon pump (IABP) was placed. Subsequently, a CT-guided endomyocardial biopsy (EMB) was performed from the right ventricular septum, which revealed numerous eosinophilic infiltrations with degranulating eosinophils, admixed lymphocytes, and a thickened endocardium (Figures 4A,B). Immunostaining for eosinophilic cationic protein showed strong staining, mainly in the endocardium and myocardial interstitium. Transmission electron microscopy also confirmed similar findings, thereby reaching the definitive diagnosis of AEM (Figures 4C,D). Intravenous methylprednisolone (1 g/day for 3 days) was initiated, followed by oral prednisolone (60 mg/day), with gradual tapering of the doses. The patient was managed in the intensive care unit for close monitoring. On day 4, serial echocardiography revealed rapid recovery of LV dysfunction and ILVWT followed by treatment with corticosteroids (Figures 2C,D and Supplementary Videos 3, 4). In parallel, the patient's condition improved dramatically, with the disappearance of eosinophilia, resulting in weaning from inotrope and IABP support. Follow-up ECG revealed the resolution of all ECG abnormalities recognized during the initial ECG (Figure 1B). On day 24, he was discharged after administration of prednisolone (20 mg/day). Thereafter, prednisolone was tapered and discontinued over 3 months. The patient remains clinically stable with normal cardiac function and no recurrence of hypereosinophilia during the 3-year follow-up. As a supplement, we present a summarized illustration of the case presentation (Supplementary Figure 1).

## Discussion

In this report, we describe a case of AEM with hemodynamic instability presenting as a low-flow HFpEF phenotype. Our case may offer several valuable clinical lessons.

First, our case, with a low-flow HFpEF phenotype, presented with recurrent presyncope. A systematic review of previous case reports and case series describing EM demonstrated that the commonest clinical manifestations were dyspnea and chest pain, with reduced LV ejection fraction of up to 35% on initial presentation (3). However, our case presented with ILVWT on admission and showed prompt complete recovery, strongly suggestive of an acute inflammatory response following AEM. The low-flow HFpEF phenotype was caused by a failure in the LV compensatory mechanism for rapidly progressive myocardial inflammation, resulting in recurrent presyncope. Thus, this case highlights the clinical significance of considering AEM as a rare cause of HFpEF and a low-flow HFpEF phenotype as a red flag in AEM.

Second, CT-guided EMB was helpful in diagnosing AEM with hemodynamic instability. Cardiovascular magnetic resonance (CMR) can characterize myocardial tissue, including inflammation, mural thrombus, and fibrosis, with a high diagnostic performance in identifying patients with stable acute myocarditis (sensitivity 81%, specificity 71%, and accuracy 79%) and is thus being considered a cornerstone for non-histological diagnosis of myocarditis (5–7).

However, CMR is contraindicated in patients with hemodynamic instability owing to long-time scanning. EMB remains the gold standard for the diagnosis of myocarditis, even in such settings. Nevertheless, the diagnosis of clinically unstable acute myocarditis remains challenging because EMB is invasive and has a low diagnostic yield due to the heterogeneous distribution of inflammatory cell infiltrates (8).

In our case, the biopsy site was not easily predictable from the ECG and echocardiographic findings. Pre-procedural CT imaging provided valuable information about the location, extent, and pattern of myocardial inflammation. Although diffuse subendocardial hypoenhancement of the LV is characteristic for EM (1), in our case, CT showed several heterogeneous nonischemic hypoenhanced areas continuous from the endocardium to the myocardium of the LV with pericardial effusion, suggesting a more intense active lesion. Subsequently, we encouraged performing an EMB from the right ventricular septum, leading to increased diagnostic accuracy. A multicenter study evaluating patients with stable acute myocarditis demonstrated that late gadolinium enhancement (LGE) on CMR can be divided into four LGE distribution patterns of LV (subepicardial layer of inferior and lateral myocardial wall, 41%; mid-anteroseptal wall, 36%; other segments, 16%; absence of LGE, 7%; respectively) (9), explaining the low diagnostic yield of blind EMB in myocarditis.

Recently, late iodine enhancement (LIE) imaging has been an emerging and promising diagnostic alternative to LGE-CMR, allowing the measurement of myocardial extracellular volume (10). In a single-center retrospective study of 20 patients with CMR-proven acute myocarditis, spectral CT with LIE was extremely useful for diagnosing acute myocarditis (82% sensitivity, 99% specificity, 94% positive predictive value, 95% negative predictive value, and 95% overall accuracy) (11). Further accumulation and analysis of data are required on the evaluation of myocarditis using CT.

Third, our case represented a quick and dramatic response to high-dose corticosteroid therapy. Although the exact mechanism of EM remains unclear, eosinophils—that have the potential to activate cardiac mast cells and release prothrombotic tissue factors and granule proteins cytotoxic to cardiomyocytes—may play a major pathological role in myocarditis (12). Furthermore, in a mouse model, eosinophil-derived IL-4 has been reported to be deeply involved in the progression from AEM to chronic inflammatory dilated cardiomyopathy (13). Therefore, treatment of AEM involves halting of eosinophilic infiltration, counteraction of inflammation, and prevention of fibrosis/thrombus formation, in addition to symptomatic treatment of heart failure. Corticosteroids have been the mainstay of treatment for AEM, and generally, AEM patients respond well to corticosteroid therapy (14). In our case, despite the rapid hemodynamic compromise, high-dose corticosteroids resulted in a rapid and dramatic recovery and normalization of cardiac structure and function in 3 days. Thus, our case underscores the paramount importance of early treatment in cases of possible AEM. The fundamental treatment of AEM is to identify the etiology of hypereosinophilia and to either avoid or treat it. The causative etiologies include hypersensitivity reactions, infections, neoplasms, immune-mediated diseases, myeloproliferative disorders including hypereosinophilic syndromes, and idiopathic or undetermined forms. Despite a thorough diagnostic evaluation, the etiology was unknown in our case.

Finally, we propose a comprehensive diagnostic algorithm consisting of clinical scenarios, biomarkers, and multimodality images that would suspect AEM during the initial diagnostic workup of HFpEF (Supplementary Figure 2) (1, 4). AEM should be strongly suspected if one or more of the red flags listed in the supplementary figure are observed in addition to ILVWT. While the combination of CMR findings and cardiac biomarkers can diagnose hemodynamically stable AEM, in patients with hemodynamic instability, as in our case, subsequent CT-guided EMB should be performed without hesitation.

## Conclusion

We describe a case of AEM, which rapidly progressed to cardiogenic shock and was successfully treated with high-dose corticosteroid therapy. EM, which can present as a low-flow HFpEF phenotype, is an uncommon but fatal inflammatory disorder, which can be cured if treated promptly during an earlier stage. Therefore, all clinicians should recognize the clinical importance of considering AEM as a rare cause of HFpEF. Given the favorable prognosis of AEM with early diagnosis and prompt treatment, clinicians should not hesitate to perform CT-guided EMB in patients suspected of having AEM with hemodynamic instability.

## Data Availability Statement

The original contributions presented in the study are included in the article/Supplementary Material, further inquiries can be directed to the corresponding author/s.

## Ethics Statement

The authors confirm that written consent for submission and publication of this case report, including the images and associated movie, has been obtained from the patient.

## Author Contributions

HY was responsible for the clinical study design and conceptualization and wrote the manuscript. HA, HY, and TT were involved in the acquisition of clinical data. KI-Y, MH, and JI analyzed and interpreted the data. All authors discussed, read, and approved the submission of this manuscript for publication.

## Conflict of Interest

The authors declare that the research was conducted in the absence of any commercial or financial relationships that could be construed as a potential conflict of interest.

## Supplementary Material

Supplementary Figure 1 Timeline of the case. Echo, echocardiography; EF, ejection fraction; ECG, electrocardiogram; LV, left ventricle; LVOT-VTI, left ventricular outflow tract-velocity time integral; IABP, intra-aortic balloon pump.

Supplementary Figure 2 Red flags that should be highly suspected of AEM during the initial diagnostic workup of HFpEF **(A)**. Diagnostic algorithm for patients suspected of having AEM **(B)**. AEM, acute eosinophilic myocarditis; HFpEF, heart failure with preserved ejection fraction; ECG, electrocardiogram; LV, left ventricle; Echo, echocardiography; GLS, global longitudinal strain; LVOT-VTI, left ventricular outflow tract-velocity time integral; CT, computed tomography; CMR, cardiovascular magnetic resonance; IHC, immunohistochemistry; EM, electron microscopy; T2WI, T2-weighted image; LGE, late gadolinium enhancement; –, negative test; +, positive test.

Supplementary Videos 1, 2 Pre-treatment transthoracic echocardiography. Transthoracic echocardiography at initial presentation in a parasternal long-axis view (Supplementary Video 1) and in a parasternal short-axis view (Supplementary Video 2) shows markedly diffuse thickening of the left ventricular wall, decreased cavity size with a preserved ejection fraction of 52%, and pericardial effusion.

Supplementary Videos 3, 4 Post-treatment transthoracic echocardiography. Post-treatment follow-up transthoracic echocardiography in a parasternal long-axis view (Supplementary Video 3) and in a parasternal short-axis view (Supplementary Video 4) shows normalization of left ventricular wall thickening and cavity size with concomitant improvement in left ventricular function with an ejection fraction of 72%. Note the improvement in pericardial effusion.

## Abbreviations

EM: Eosinophilic myocarditis
AEM: Acute eosinophilic myocarditis
ECG: Electrocardiogram
ILVWT: Increased left ventricular wall thickness
LV: Left ventricle
HFpEF: Heart failure with preserved ejection fraction
CT: Computed tomography
IABP: Intra-aortic balloon pump
EMB: Endomyocardial biopsy
CMR: Cardiovascular magnetic resonance
LGE: Late gadolinium enhancement
LIE: Late iodine enhancement.

## Nomenclature

### Resource Identification Initiative

To take part in the Resource Identification Initiative, please use the corresponding catalog number and RRID in your current manuscript. For more information about the project and for steps on how to search for an RRID, please click here.

### Life Science Identifiers

Life Science Identifiers (LSIDs) for ZOOBANK registered names or nomenclatural acts should be listed in the manuscript before the keywords with the following format:

urn:lsid: <Authority>: <Namespace>: <ObjectID>[: <Version>]

For more information on LSIDs please see Inclusion of Zoological Nomenclature section of the guidelines.
