# Supplementary figures and images for: Case Report: Acute Eosinophilic Myocarditis With a Low-Flow Heart Failure With Preserved Ejection Fraction Phenotype
Source: Front Cardiovasc Med. 2021 Jun 23;8:678973. doi: 10.3389/fcvm.2021.678973 (PMC8260850; doi:10.3389/fcvm.2021.678973)

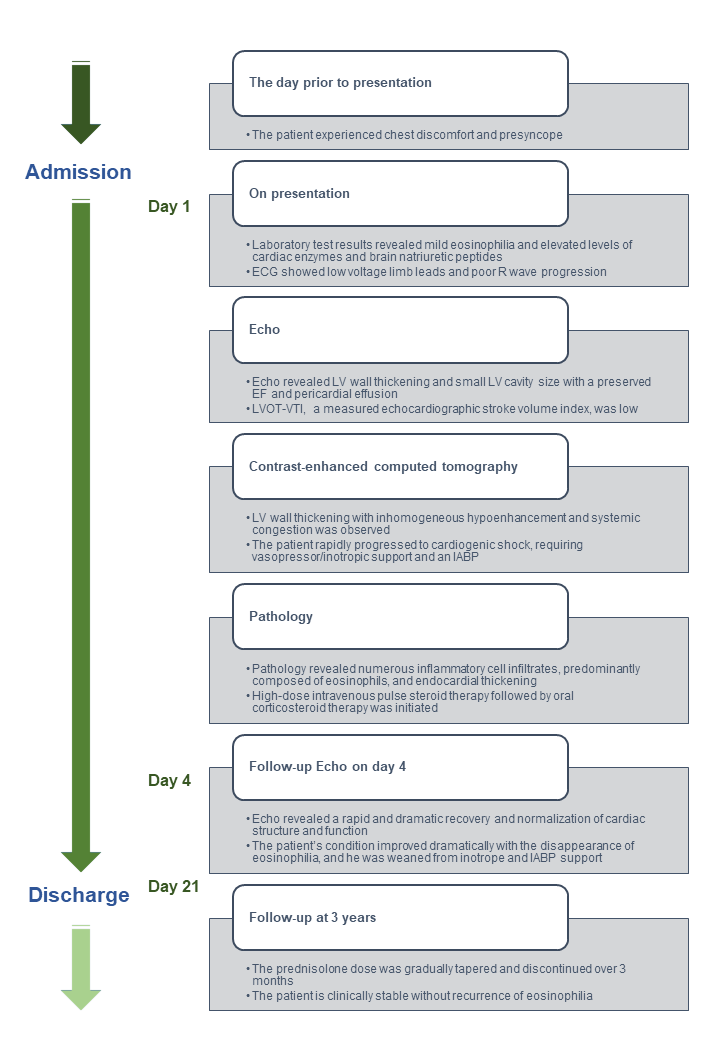

Supplement: Supplementary Figure 1 — Timeline of the case. Echo, echocardiography; EF, ejection fraction; ECG, electrocardiogram; LV, left ventricle; LVOT-VTI, left ventricular outflow tract-velocity time integral; IABP, intra-aortic balloon pump. [file Image_1.TIF]

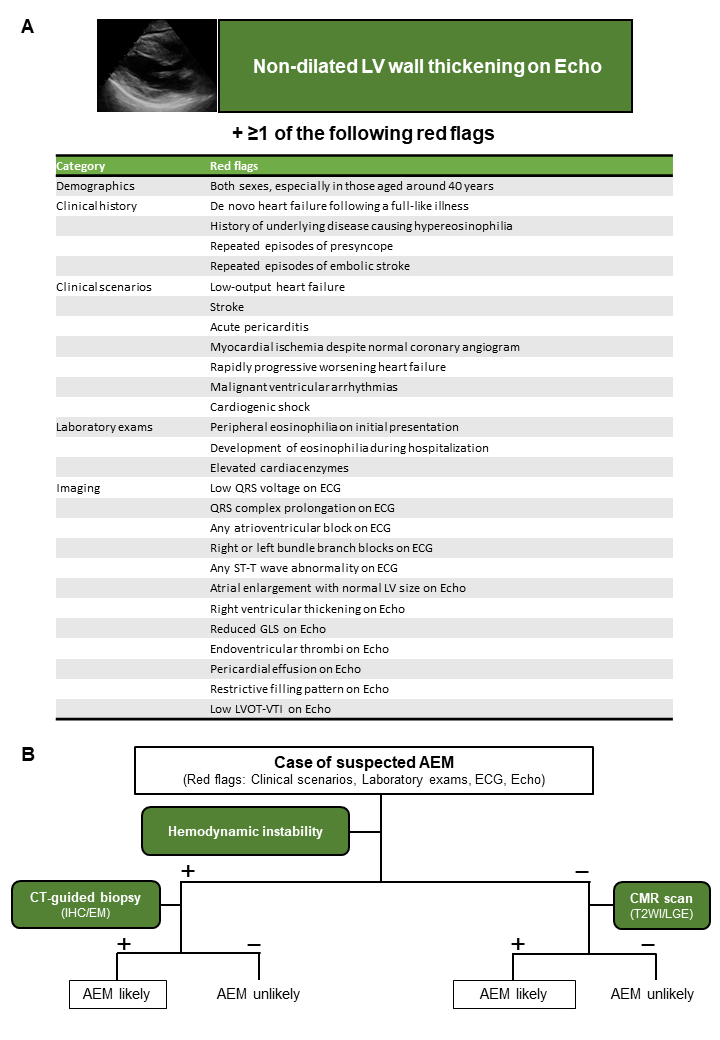

Supplement: Supplementary Figure 2 — Red flags that should be highly suspected of AEM during the initial diagnostic workup of HFpEF (A). Diagnostic algorithm for patients suspected of having AEM (B). AEM, acute eosinophilic myocarditis; HFpEF, heart failure with preserved ejection fraction; ECG, electrocardiogram; LV, left ventricle; Echo, echocardiography; GLS, global longitudinal strain; LVOT-VTI, left ventricular outflow tract-velocity time integral; CT, computed tomography; CMR, cardiovascular magnetic resonance; IHC, immunohistochemistry; EM, electron microscopy; T2WI, T2-weighted image; LGE, late gadolinium enhancement; –, negative test; +, positive test. [file Image_2.TIF]
